# LONELINESS ACROSS THE LIFE COURSE; LIFE STORY INTERVIEWS WITH MENTAL HEALTH SERVICES USERS

**DOI:** 10.1093/geroni/igac059.614

**Published:** 2022-12-20

**Authors:** Roger O'Sullivan, Annette Burns, Gerry Leavey, Jeannette Golden, Dermot Reilly, Brian Lawlor

**Affiliations:** Institute of Public Health in Ireland, Belfast/Dublin, Ireland; Institute of Public Health, Dublin, Dublin, Ireland; Ulster University, Belfast, Northern Ireland, United Kingdom; Saint James' Hospital, Dublin, Dublin, Ireland; Saint James' Hospital, Dublin, Dublin, Ireland; Trinity College Dublin, Dublin, Dublin, Ireland

## Abstract

**Introduction:**

The complexity of loneliness and its negative impact on our health and wellbeing is well established. However, the qualitative experience of loneliness over the life course is poorly understood. Method: This presentation, based on 18 life story interviews, with a sample of older adults, who were attending a mental health service and objectively defined as lonely, provides an insight into the personal experiences of loneliness as well as the situations and factors associated with loneliness across the life course.

**Results:**

The analysis identified three distinct typologies of loneliness; those who experienced (1) chronic loneliness across their life (2) those whose loneliness became chronic after a transition e.g. bereavement (3) those whose loneliness remained situational/transitional.

**Conclusions:**

The insights are important to inform both general loneliness services and policy as well as specialist mental health services and training. The presentation demonstrates the importance of a life course approach to addressing and understanding loneliness.

